# Disease flare of ankylosing spondylitis presenting as reactive arthritis with seropositivity: a case report

**DOI:** 10.1186/1752-1947-6-60

**Published:** 2012-02-14

**Authors:** EM Manoj, MK Ragunathan

**Affiliations:** 1 Ward 42, National Hospital of Sri Lanka, Colombo, Sri Lanka

## Abstract

**Introduction:**

Concurrent rheumatoid factor seropositivity is occasionally detected in ankylosing spondylitis and often causes confusion in clinical routine. Overlap between various seronegative arthritides is a known but uncommon association. Differentiation of spondyloarthropathy from rheumatoid arthritis is important, since the natural history, complications, treatments and prognosis of the two diseases differ significantly.

**Case presentation:**

Here, we report the case of a 47-year-old Sri Lankan man who had a long history of intermittent joint pains worsening following a recent episode of self-resolving non-bloody diarrhea. Subsequently, he developed a skin rash suggestive of keratoderma blenorrhagica and circinate balanitis. He had classical radiological evidence of ankylosing spondylosis (previously undiagnosed) associated with human leukocyte antigen B27 antigen, but was positive for rheumatoid factor.

**Conclusions:**

A disease flare of ankylosing spondylitis prompted by a minor diarrheal illness showing well documented features of reactive arthritis is remarkable. The prognostic implications of seropositivity in spondyloarthritis are discussed.

## Introduction

Estimates of the prevalence of spondyloarthritis range from 1% to 2% of the population, and are similar to the prevalence of rheumatoid arthritis (RA). The lack of any association with autoantibodies including rheumatoid factor (hence the term 'seronegative'), an increased incidence of ankylosing spondylitis (AS) in the whole group and the association with human leukocyte antigen (HLA)-B27 are the features common to this group of diseases [1]. The prognostic implications of early administration of disease-modifying drugs in RA, but not in spondyloarthropathy, are well established. The unusual presentation of reactive arthritis as a flare up of AS following a minor diarrheal illness associated with seropositivity prompted us to report this case.

## Case presentation

A 47-year-old man who worked as a driver was admitted to our facility with a three-week history of worsening peripheral joint pains following an episode of watery diarrhea. The joint pains mainly involved the right side of his shoulder girdle, right wrist, right second toe and right Achilles' tendon. He had morning stiffness in the involved joints lasting for around one hour. He did not have significant backache, urinary symptoms or red eyes. In the past 20 years he had experienced intermittent episodes of joint pains and swelling involving his right knee joint, right elbow joint and right shoulder joint. Those episodes were not preceded by gastrointestinal or urinary infections and he denied any episodes similar to his current presentation. He had no history to suggest inflammatory bowel disease or psoriasis.

**Figure 1:**
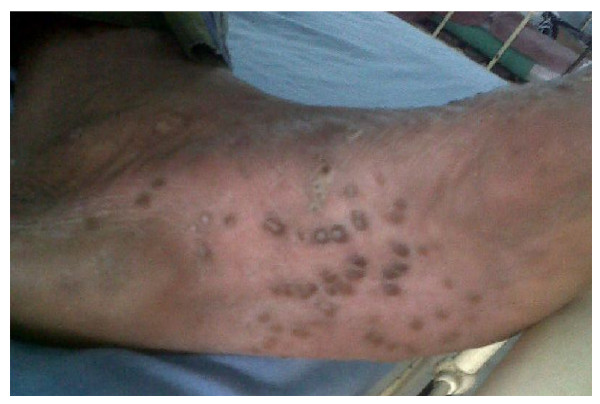
**Palmoplantar pustulosis, suggestive of keratoderma blenorrhagica**.

A physical examination revealed an averagely built man with a stiff posture. Our patient was not dyspneic but his chest expansion (1.7 cm) was impaired. Swelling and tenderness of the right side of the shoulder girdle was associated with marked restriction of the anterior and lateral flexion of spine. There was a demonstrable right-sided Achilles tendinitis. His swollen, tender right second toe was suggestive of 'sausage finger'. He had a non-scaly, reddish brown discrete papular eruption on his palms and soles suggestive of keratoderma blenorrhagica (Figure 1). Evidence for lung fibrosis or cardiac valvular involvement was absent. He developed circinate balanitis some weeks later.

Laboratory investigations revealed mild neutrophil leukocytosis with markedly elevated inflammatory markers (erythrocyte sedimentation rate (ESR) = 115 mm/hour, C-reactive protein (CRP) = 96 mg/dL). A test for rheumatoid factor was positive (64 IU/mL; latex method, cut-off < 8 IU/mL) but an anti-cyclic citrullinated peptide (CCP) antibody test (medical image analysis method) was negative. A urine microscopy study and routine culture tests were also negative. A stool culture test was also negative. An enzyme-linked immunosorbent assay (ELISA) test for HIV, VDRL test and *Treponema pallidum *particle agglutination (TPPA) assay were also all negative. A test for human leukocyte antigen (HLA)-B27 was also positive (lymphocytotoxicity method).

**Figure 2:**
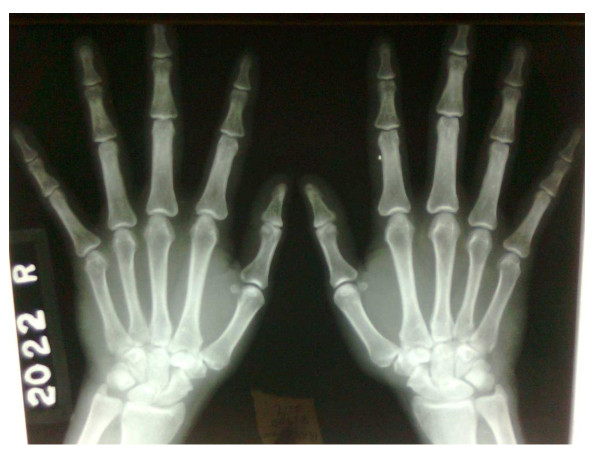
**X-ray of hands**.

**Figure 3:**
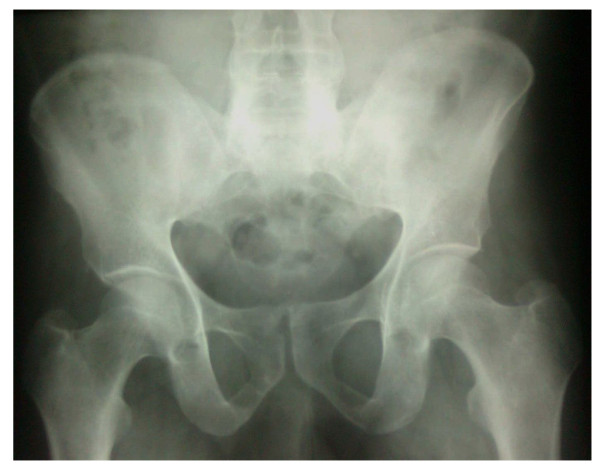
**Advanced symmetrical sacroiliitis**.

**Figure 4:**
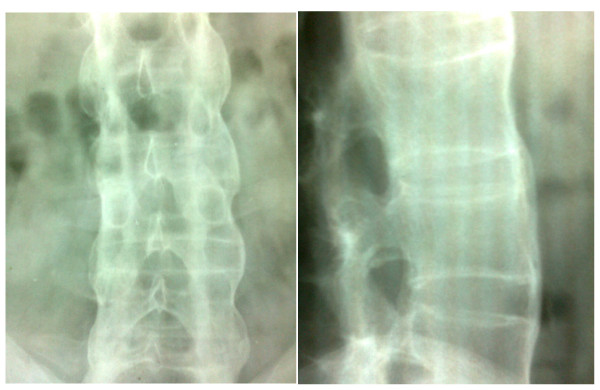
**'Bamboo spine' with widespread bridging syndesmophytes**.

**Figure 5:**
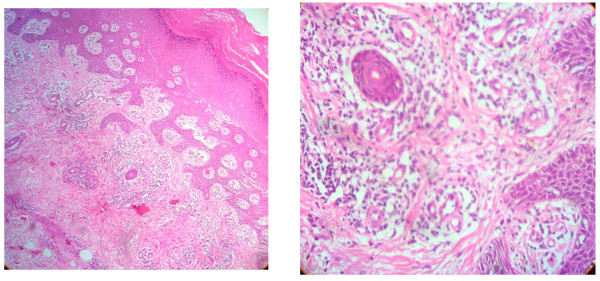
**Biopsy of skin lesions**.

Radiography of his hands (Figure 2), wrists, right shoulder joint and foot did not reveal any erosive changes. Both sacroiliac joints were markedly sclerosed (Figure 3) and the typical bamboo spine with widespread bridging syndesmophytes (Figure 4) was seen. Biopsy of his skin lesions (Figure 5) revealed hyperkeratosis forming a thin horny layer and psoriasiform hyperplasia, and papillary dermis showed chronic inflammatory cell infiltrates and superficial dermal edema.

Treatment with systemic steroids and non-steroidal anti-inflammatory drugs (NSAIDs) was not successful. Consequently our patient was started on sulfasalazine. Intra-articular injections of steroids and sulfasalazine lead to remission of his current episode. He managed to re-engage in his occupation as a driver with satisfactory improvement of joint symptoms while on sulfasalazine therapy.

## Discussion

To the best of our knowledge, the case of our patient is unique in the clinical, radiological and serological associations of oligoarthritis aggravated following a diarrheal episode. Keratoderma blenorrhagica, circinate balanitis, ankylosing spondylitis, presence of HLA-B27 antigen and seropositivity are unorthodox associations [2]. Though 3% of the normal population may have rheumatoid factor positivity, its occurrence in this case is less likely to be coincidental, considering the high titer of rheumatoid factor.

Peripheral oligoarthritis aggravated following a diarrheal episode followed by keratoderma blenorrhagica and circinate balanitis suggested a diagnosis of reactive arthritis [3], whereas only one-third of patients show the complete triad of arthritis, conjunctivitis and urethritis. The classical radiological features of more symmetrical bilateral sacroiliitis and typical 'bamboo spine' in our patient, associated with limitation of movement of the lumbar spine in the sagittal and lumbar planes, was adequate to diagnose AS according to the modified New York criteria.

Possible overlap with psoriatic arthritis was also considered for two reasons. Histologically, his skin eruption shared some features favoring palmoplantar psoriasis rather than keratoderma blenorrhagica [4,5]. Isolated inflammation of an entire toe giving the appearance of a 'sausage digit' may occur in both reactive and psoriatic arthritis as well. However, psoriatic arthritis is usually gradual in onset, the arthritis tends to affect primarily the upper extremities, and there is far less associated periarthritis.

Despite seropositivity, the joint involvement and radiological evidence favors a diagnosis of AS rather than RA. Some practical difficulties do exist in the differentiation as up to half of patients with AS have peripheral joint involvement during their disease and the spine may be more or less spared [6]. There are only a few other reported cases where spondyloarthritis has been linked to seropositivity ([7,8] see also Table 1). We searched PubMed using the keywords 'ankylosing spondylitis' or 'reactive arthritis' with 'seropositivity' appearing anywhere in the article and repeated the same search (without time limits to the search) in Google Scholar (Table 1). Poor response to NSAIDs and remarkable improvement of joint symptoms with disease-modifying anti-rheumatic drugs (DMARDs) in a patient with classical evidence of spondyloarthritis in the context of seropositivity is a platform for discussion.

**Table 1:** Summary of literature review of patients with simultaneous occurrence of rheumatoid arthritis and ankylosing spondylosis

Reference	Age	Gender	HLA-B27	Rheumatoid factor	Erosions	Sacroiliitis
7	64	M	+	1/640	+	Bilateral
7	68	M	NA	1/640	+	Bilateral
7	68	F	+	1/640	-	Bilateral
7	54	M	+	1/2560	+	Bilateral
7	67	M	+	1/80	+	Bilateral
7	75	F	-	1/160	+	Bilateral
7	58	F	+	1/320	+	Bilateral
7	62	M	+	1/320	+	Bilateral
7	44	M	+	1/2048	+	Bilateral
8	42	M	NA	-	+	Bilateral

HLA = human leukocyte antigen; NA = not available.

## Conclusions

Disease flare of AS prompted by a minor gastrointestinal infection showing well documented features of reactive arthritis is remarkable. A possible explanation for the associations of this case, though less likely, is that one of the diseases occurred by chance in a patient already suffering from the others. However the satisfactory response to disease modifying drugs in the background of seropositivity has an effect on overall prognosis.

## Consent

Written informed consent was obtained from the patient for the publication of this case report and any accompanying images. A copy of the written consent is available for review by the Editor-in-Chief of this journal.

## Competing interests

The authors declare that they have no competing interests.

## Authors' contributions

EMM analyzed and interpreted the data from our patient regarding the uncommon presentation of rheumatological diseases. The literature review and corrections were performed by MKR. Both authors read and approved the final manuscript.
